# Treatment of restless legs syndrome by acupuncture combined with medicine based on pathophysiological mechanism

**DOI:** 10.3389/fmed.2026.1785644

**Published:** 2026-05-20

**Authors:** Rujia Liu, Qi Wang

**Affiliations:** 1 Heilongjiang University of Chinese Medicine, Harbin, Heilongjiang, China; 2 Shiyan Hospital of Traditional Chinese Medicine, Shíyàn, Hubei, China

**Keywords:** acupuncture combined with medication, dopamine system, iron metabolism, pathophysiological mechanism, RLS, treatment strategy

## Abstract

Restless legs syndrome (RLS) is a neurological disorder characterized by bilateral discomfort in the lower extremities, intense urge to move, and exacerbation of symptoms during the night. Its pathogenesis is closely associated with dysfunctions in the dopaminergic system, abnormal iron metabolism, and genetic predisposition. RLS frequently co-occurs with conditions, such as chronic kidney disease and iron deficiency anemia, significantly impairing patients’ sleep quality and daytime functioning. This review systematically examines the definition, classification, epidemiological features, and diagnostic criteria of RLS, and provides an in-depth analysis of its core pathophysiological mechanisms. Furthermore, it offers a comprehensive overview of current pharmacological treatments, including dopamine receptor agonists, α2δ ligands, and iron supplementation, as well as non-pharmacological interventions, such as exercise, transcutaneous direct current spinal cord stimulation, and acupuncture. The synergistic effects of combined acupuncture and pharmacotherapy were explored, highlighting mechanisms involving neural regulation, iron metabolism modulation, and immune function enhancement, along with their clinical efficacy. Additionally, the review addresses ongoing controversies in treatment, including the stability of therapeutic outcomes and unclear mechanisms underlying interventions. Finally, it outlines prospective research directions emphasizing multidisciplinary collaboration, precision medicine approaches, and multicenter clinical trials, aiming to provide a theoretical framework and practical foundation for the individualized diagnosis and management of RLS.

## Introduction

Restless legs syndrome (RLS; Willis–Ekbom disease) is a common sensorimotor disorder defined by an urge to move the legs but with unpleasant sensations that may worsen at rest, show a circadian pattern (particularly during the evening or at night), and are partially or completely relieved by movement (1). Apart from the leg discomfort, RLS is clinically important because it disrupts sleep due to co-occurrence with periodic limb movements and degrades daytime functioning and quality of life (1). However, due to lack of research on this topic, epidemiologic estimates vary across case definitions and sampling frames. A recent systematic meta-analysis on adult populations reported an overall pooled prevalence of ∼3%, with higher prevalence among women and older individuals (2). Importantly, an expert consensus management algorithm indicated that ∼1.5%–2.7% of the population experiences more frequent and distressing symptoms (≥2 times/week with at least moderate distress) that typically require active treatment (3).

Recent investigations in this field have improved our mechanistic understanding on RLS, with evidence implicating dopaminergic dysfunction, brain iron deficiency, and additional signaling pathways that may affect sensory symptoms and motor hyperexcitability (1, 4). In addition, research on human genetics has advanced rapidly, with the current largest genome-wide meta-analyses now showing a far larger set of risk loci than previously recognized (including > 100 loci), enabling the identification of significant pathways and risk prediction (5). However, despite these findings, knowledge on dopamine biology, iron trafficking and polygenic risk remains not yet fully integrated into a unified, clinically actionable pathophysiological model that may effectively guide individualized therapy across heterogeneous patient subtypes (1, 4, 5).

Moreover, current pharmacology options remain constrained due to limited literature on long-term tolerability and safety. Although dopamine agonists have been shown to be initially effective, long-term use can cause symptoms to worsen again, such as making the symptoms to start earlier in the day, becoming stronger, or spreading to other body parts, and because of this risk and other side effects, current guidelines place more emphasis on α2δ ligands and careful evaluation and correction of iron deficiency (1, 6, 7). Meanwhile, there has been an increasing interest in acupuncture and integrative regimens, with pooled analyses suggesting potential symptom improvement. Nevertheless, the evidence base remains highly heterogeneous and fragmented across small trials, crossover designs, case series, and variable point prescriptions, dosing, comparators, and outcomes, limiting certainty and generalizability (8, 9). In this regard, we designed this review to synthesize current recent evidence linking RLS pathophysiology (dopamine–iron–genetic architecture) to treatment decision-making, with particular focus on acupuncture combined with pharmacotherapy, and to identify practical gaps to inform standardized protocols and future rigorous multicenter trials. The details of our literature search and review methodology are provided in Supplementary File 1.

## Diagnosis and classification of RLS

**FIGURE 1:**
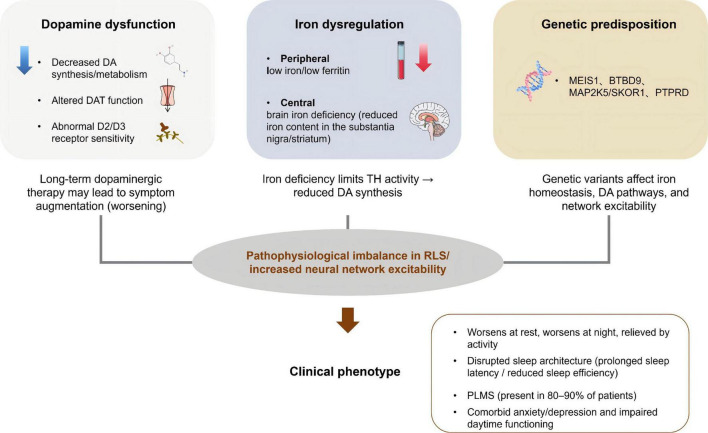
Diagnostic pathway and treatment decision-making for restless legs syndrome (RLS). This figure outlines the diagnostic and treatment decision-making framework for RLS. The process begins with symptom screening (four key features: leg discomfort, worse at rest, worse at night, relieved by movement). If IRLSSG/ICSD-3 criteria are met, differential diagnosis is performed to exclude mimics such as peripheral neuropathy, vascular disorders, musculoskeletal pain, and medication-related causes. Secondary causes (e.g., iron deficiency, pregnancy, CKD/dialysis, PD) are screened. Based on classification (primary vs. secondary), severity and impact on daily life are assessed using the IRLSRS, and objective tests (iron status, PSG, imaging) are performed. Treatment decisions are made based on subtype, severity, ferritin levels, PLMS, comorbidities/risks. RLS: restless legs syndrome; IRLSSG: International Restless Legs Syndrome Study Group; ICSD-3, International Classification of Sleep Disorders, Third Edition; PSG, polysomnography; PLMS, periodic limb movements in sleep; PD, Parkinson’s disease; CKD, chronic kidney disease. Created with Figdraw.com.

In Western medicine, RLS is defined as a sensorimotor disorder marked by an urge to move the legs with uncomfortable sensations and relief with movement, as well as exclusion of alternative medical conditions that could account for the symptoms and symptom occurrence at a frequency of at least three times per week over a minimum duration of 3 months (10). The diagnostic workflow emphasizes careful clinical history plus exclusion of common “mimics” and contributors (e.g., iron deficiency, renal disease, medications) (Figure 1), because these factors substantially affect management choices and long-term outcomes (11). RLS is classified into two categories: primary (idiopathic) and secondary. Primary RLS is predominantly associated with genetic predispositions, whereas secondary RLS frequently occurs in individuals with chronic kidney disease (CKD), iron deficiency anemia, pregnancy, or Parkinson’s disease (12, 13).

From a traditional Chinese medicine (TCM) perspective, RLS is not approached as a single fixed disease defined by one diagnostic checklist; instead, it is commonly considered to be a cluster of symptoms that requires pattern identification to guide individualized therapy. In current integrative clinical studies, this difference is visible in the outcome and classification, whereby investigators report both biomedical severity and TCM symptom scores, reflecting an attempt to use pattern-based assessment alongside Western disease severity grading (14).

Its classification in TCM is based on patterns rather than etiologic categories to link the patient’s symptom quality, timing, constitution and accompanying systemic features to a specific treatment principle and formula strategy. This also provides a way to integrate acupuncture and moxibustion, where therapy is delivered through meridian- and point-based protocols selected to target symptom clusters and sleep-related disturbances. In recent integrative clinical research, there has been a trend to first diagnose and assess RLS using Western severity scores [e.g., International Restless Legs Syndrome Rating Scale (IRLS)], and then add TCM-specific characterization (e.g., TCM symptom scores and pattern-informed prescriptions) to stratify patients and tailor interventions (14, 15). For instance, a recent comparative clinical study measured both IRLS severity and TCM symptom scores when evaluating a TCM decoction added to conventional pharmacotherapy, illustrating how TCM classification can be merged with Western practice, which resulted in ameliorating patients’ clinical symptoms, reducing RLS severity, and improving their quality of life and sleep quality (14). In addition, Chen et al. (15) diagnosed patients with RLS using standard Western criteria and used IRLSRS, the Insomnia Severity Index and heart-rate variability on treatment outcomes between an experiment (acupuncture) and a control group. They found that acupuncture improved RLS symptoms, insomnia scores, and autonomic markers more than control treatment, demonstrating a straightforward integrative model: Western criteria for diagnosis and evaluation, TCM methods for treatment delivery. Thus, presenting RLS through this dual structure helps standardize comparisons across heterogeneous studies and clarifies why treatment choices differ between modalities.

## Pathological mechanism of RLS

**FIGURE 2:**
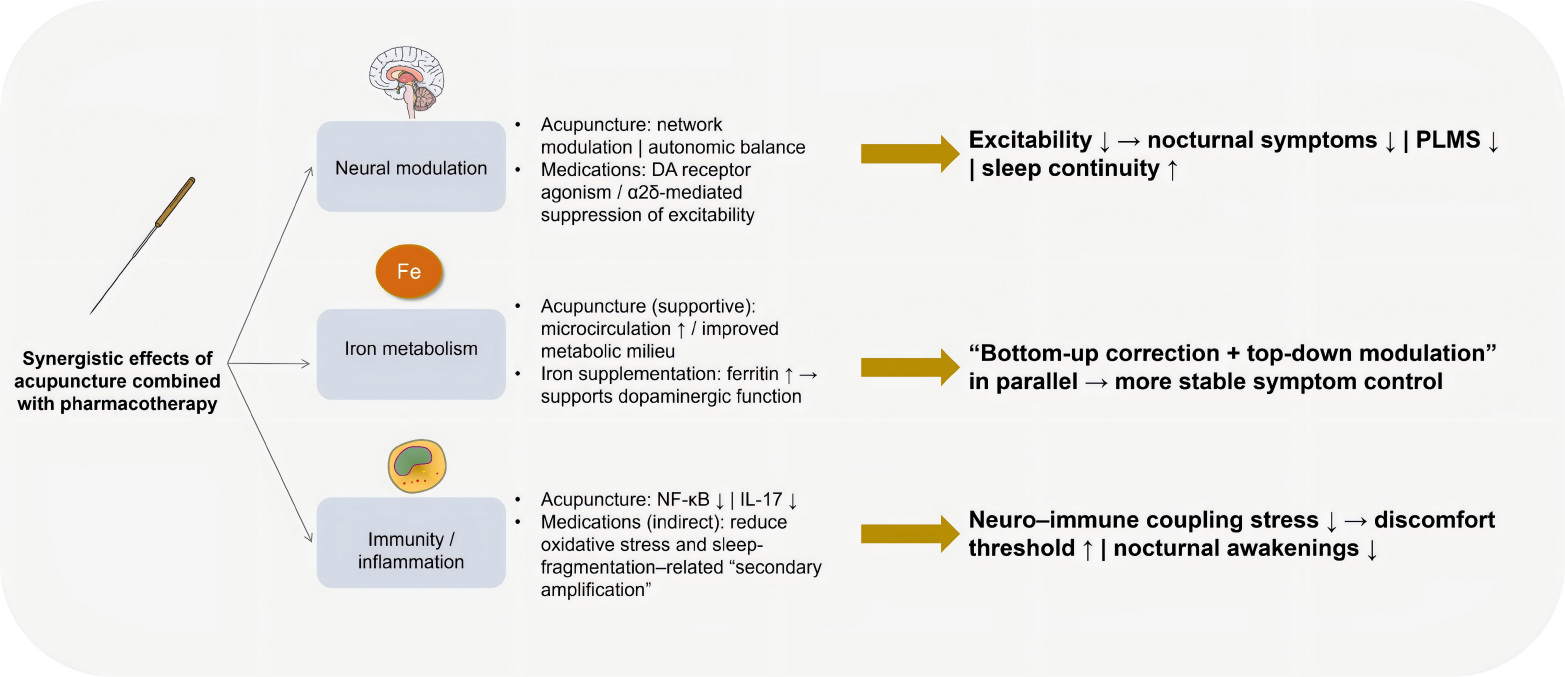
Three-axis mechanistic framework of RLS and the resulting clinical phenotype. The figure summarizes three core axes implicated in RLS—dopaminergic dysfunction, iron dysregulation, and genetic predisposition—that converge on increased neural network excitability/pathophysiological imbalance. The downstream phenotype includes worsening at rest and at night with relief by activity, disturbed sleep architecture, frequent PLMS, and comorbid mood symptoms with impaired daytime functioning. Augmentation with long-term dopaminergic therapy is also highlighted. RLS, restless legs syndrome; DA, dopamine; DAT, dopamine transporter; D2/D3, dopamine D2/D3 receptor; TH, tyrosine hydroxylase; PLMS, periodic limb movements in sleep; SN, substantia nigra. Created with Figdraw.com.

The pathophysiology of RLS can be described through three major mechanistic domains across biomedical studies: disturbances in dopaminergic neurotransmission, abnormalities in iron metabolism, and genetic susceptibility. These mechanisms form the biological foundation upon which current Western treatments and emerging integrative interventions, including acupuncture, have been developed (Figure 2).

## Dopaminergic dysfunction

Dopaminergic dysregulation remains the most widely supported mechanism. Imaging studies using 99mTc-TRODAT-1 SPECT have shown that striatal dopamine transporter uptake is approximately 20%–30% lower in patients with RLS compared with healthy controls, and these reductions correlate with symptom severity (16). Reduced cerebrospinal fluid concentrations of dopamine metabolites, such as homovanillic acid, provide additional evidence of impaired dopamine synthesis (17). Experimental models further support this pathway: Btbd9 knockout mice exhibit decreased striatal D2 receptor expression together with RLS-like motor activity and increased nocturnal movement (18). Clinically, the efficacy of dopamine agonists also reinforces the central role of dopaminergic signaling in symptom generation, although long-term treatment may lead to receptor desensitization and augmentation in a proportion of patients (19, 20). These dopaminergic abnormalities are highly relevant to integrative therapy research. Many acupuncture protocols target meridians associated with neuromodulatory regulation, and several mechanistic studies suggest that acupuncture can modulate central dopaminergic tone, providing a physiologic explanation for its clinical effects when used alone or in combination with medication.

## Iron deficiency and disordered iron metabolism

Iron is essential for dopamine synthesis because it serves as a cofactor for tyrosine hydroxylase. Clinical studies report reduced serum ferritin levels in RLS patients, and symptom severity is often inversely related to ferritin concentration (12, 21). Importantly, iron deficiency is not limited to peripheral circulation. Magnetic resonance imaging studies demonstrate reduced iron content in the substantia nigra and striatum, even in patients with normal serum iron, indicating a specific deficit in brain iron availability (22), which contributes to impaired dopamine synthesis, increased neuronal excitability, and a greater likelihood of periodic limb movements during sleep (23, 24). The iron pathway directly supports the rationale for integrative therapy. Western guidelines recommend iron repletion as an important strategy, whereas in TCM, formulas such as Danggui Buxue Decoction are traditionally prescribed for patterns interpreted as “blood deficiency,” fatigue, and related symptom clusters that clinically overlap with iron-deficiency–associated RLS. Therefore, understanding the shared mechanistic link through iron metabolism reinforces why combined approaches may offer additive benefits.

## Influence of genetic factors on RLS

Genetic factors have been shown to significantly contribute to the morbidity of RLS, as evidenced by genome-wide association studies that have identified multiple loci linked to increased RLS risk. Among these, MEIS1 is one of the most prominent risk genes, and its polymorphisms have been reported to show a strong association with RLS susceptibility (25). For instance, research involving patients diagnosed with both migraine and RLS demonstrated a significant correlation between the rs2300478 locus of MEIS1 and RLS risk, with a notably stronger effect observed in individuals experiencing episodic migraine (25). Furthermore, variations in MEIS1 have been linked to the severity of RLS symptoms, with carriers of the risk allele exhibiting more pronounced clinical manifestations (25). In addition, BTBD9 polymorphisms have been shown to correlate with lower ferritin levels and increased periodic limb movements (26). These genetic models strengthen the concept that RLS arises via shared mechanisms, such as the disruption of iron homeostasis and dopaminergic neurotransmission, underscoring the polygenic nature of RLS. They also help explain why Western pharmacologic interventions, such as dopamine agonists, α2δ ligands, and iron supplementation, and non-pharmacologic or integrative modalities, including acupuncture, can influence symptom expressions by acting on overlapping neurochemical, metabolic and neurophysiological targets. Strategizing therapies based on this shared mechanistic background indicates that different modalities do not operate in isolation but rather address distinct aspects of the same underlying pathophysiology. On this basis, the next section reviews therapeutic strategies for RLS, with particular focus on how pharmacologic, non-pharmacologic, and integrative acupuncture-based interventions may be combined or sequenced in a rational, mechanism-informed manner.

## Therapeutic strategies for RLS

**FIGURE 3:**
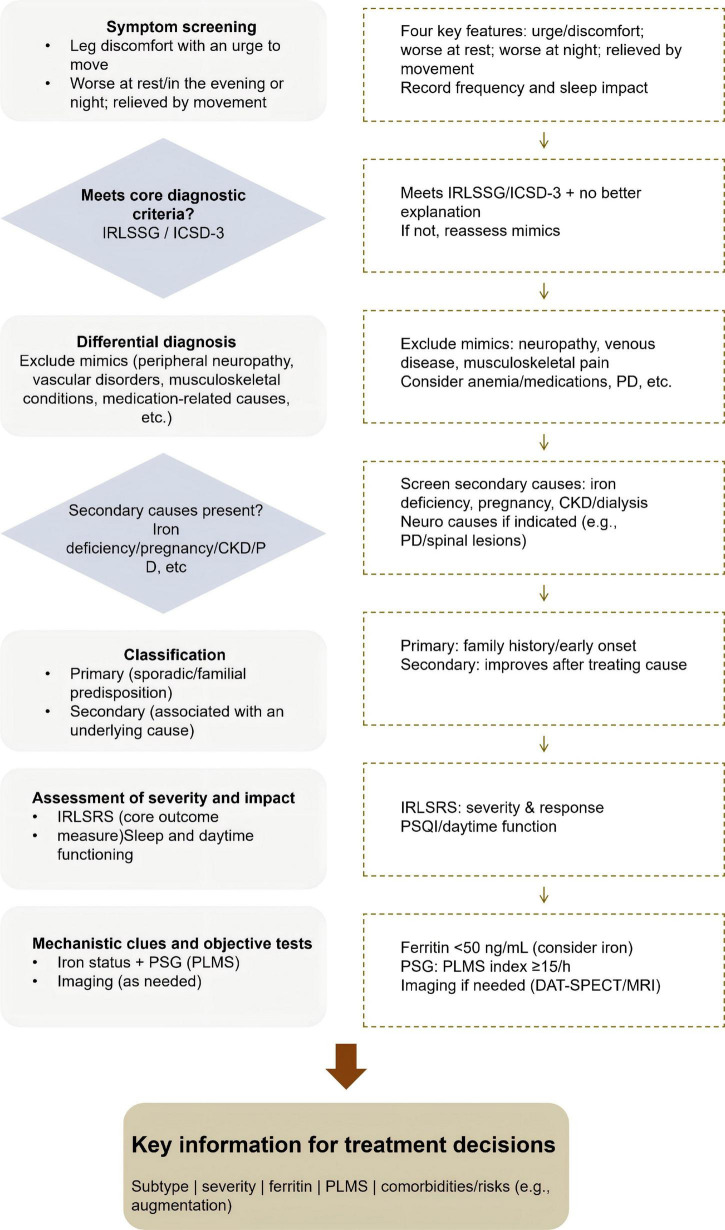
Pharmacologic and non-pharmacologic treatment of restless legs syndrome (RLS). This figure summarizes pharmacologic and non-pharmacologic treatment strategies for RLS and their shared treatment goals. Pharmacologic treatments include DA receptor agonists (symptom relief, monitor for augmentation), α2δ ligands (improve sleep for sleep-disturbed phenotype), and iron supplementation (for iron-deficient patients). Non-pharmacologic treatments include exercise interventions, neuromodulation, lifestyle modifications, and acupuncture/massage/phototherapy. The shared treatment goals are: symptom relief, improved sleep, reduced periodic limb movements in sleep (PLMS), and enhanced daytime functioning. tsDCS: transcutaneous spinal direct current stimulation.

The treatment of RLS involves pharmacologic, non-pharmacologic, and increasingly, integrative approaches (Figure 3). Thus, a mechanism-informed strategy is essential for selecting and sequencing these interventions, given the roles of dopaminergic dysfunction, altered iron metabolism, and neural hyperexcitability in RLS pathophysiology.

## Pharmacologic therapy

Dopamine agonists remain a key therapeutic class and include pramipexole, ropinirole, and rotigotine. A recent meta-analysis demonstrates 60%–70% symptom improvement with pramipexole, together with reductions in standardized severity scores (19). The rotigotine transdermal patch has been shown to provide continuous dopaminergic stimulation and clinically meaningful IRLS reductions after 12 weeks of treatment (27). However, long-term exposure may be associated with a 10%–20% risk of augmentation, such as earlier symptom recurrence onset, stronger symptoms, or spread to additional body regions, which limits their sustained use in some patients (20).

α2δ ligands such as gabapentin and pregabalin have been shown to provide an alternative first-line option, especially for individuals with sleep disturbance or intolerance to dopamine agonists. Gabapentin has been shown to outperform levodopa in hemodialysis-associated RLS (28), while pregabalin achieves a 50%–60% response rate and improves sleep and daytime functioning (29). Moreover, iron supplementation is recommended when ferritin levels fall below 50 ng/mL. Oral or intravenous formulations both reduce symptom severity, with parenteral iron offering more rapid correction in patients with poor absorption or intolerance (24). In addition, since disordered brain iron metabolism contributes to dopaminergic dysfunction, correction of iron deficiency is considered a pivotal part of the treatment plan.

## Non-pharmacologic interventions

Non-drug therapies help address neural hyperexcitability, autonomic imbalance, and sleep disruption. Aerobic exercise has demonstrated sustained improvements in RLS symptom severity, including in patients with ESRD-associated RLS (30, 31). Transcutaneous spinal direct current stimulation (tsDCS) has been shown to reduce symptom burden and enhance sleep quality, likely by modulating sensorimotor pathway excitability (32).

Moreover, massage therapy has been shown to provide symptomatic benefit, with improvements in severity and sleep quality after structured foot or lower-limb massage programs (33). In addition, acupuncture, evaluated in multiple randomized controlled trials, has been associated with reductions in IRLS scores and improvements in sleep-related outcomes. Furthermore, standardized acupoint protocols (e.g., ST36, SP6, BL60, GB41) have been shown to reduce nocturnal activity, early sleep activity, and IRLS scores by approximately 9–10 points over 4–6 weeks (9, 34). Additionally, mechanistic studies suggest modulation of autonomic balance, increased regional blood flow, and changes in cortical activity patterns (35, 36). Lastly, some additional modalities such as acupoint injection, electroacupuncture and near-infrared acupoint therapy have shown measurable improvements in IRLS scores, sleep quality, and pain parameters (15, 37, 38).

## Basic theory of RLS treated by acupuncture combined with medicine

The theoretical basis for treating RLS with acupuncture combined with medication is based on several complementary biological mechanisms, including modulation of neural activity, improvement of local microcirculation, and regulation of immune and autonomic functions. Findings from controlled studies indicate that acupuncture can influence numerous physiological domains associated with RLS symptom generation. For instance, a randomized controlled trial using standardized acupuncture at commonly used acupoints such as GB41, BL60, ST36, and SP6 reported significant reductions in nocturnal activity and early sleep activity. After 6 weeks of treatment, the patients had an average decrease of 9.45 points on the IRLSRS scale, with no notable adverse events, suggesting that these acupoint combinations can exert direct effects on sleep–wake regulation and sensory–motor symptoms (9, 34).

Subsequent mechanistic investigations have shown that acupuncture may also influence central autonomic pathways. Studies using repetitive transcranial magnetic stimulation have demonstrated that changes in the amplitude of low-frequency fluctuations in the sensorimotor cortex correspond closely to reductions in IRLSRS scores, indicating that such interventions may modulate cortical excitability, including acupuncture, and reduce symptoms through central neuromodulation rather than only peripheral mechanisms (35). Near-instantaneous symptomatic relief has also been observed with acupoint injection, and despite being small case series, some have reported that injections of normal saline at GB41, BL60, ST36, and SP6 produced rapid improvements in leg discomfort and motor restlessness, with marked decreases in Visual Analog Scale (VAS) scores (37). Moreover, related mechanistic studies have shown that acupuncture may trigger endogenous opioid activation, enhance local blood flow, and improve tissue oxygenation and metabolic activity, all of which are physiologically plausible pathways for symptom relief in RLS (36). Similarly, near-infrared light therapy on the lower-limb acupoints for 12 weeks has been shown to reduce International Restless Legs Syndrome Rating Scale (Revised Version) (IRLSRS) scores by 3.8 points in hemodialysis patients, suggesting that improving microcirculation and nerve conduction contributes meaningfully to clinical response (38).

**FIGURE 4:**
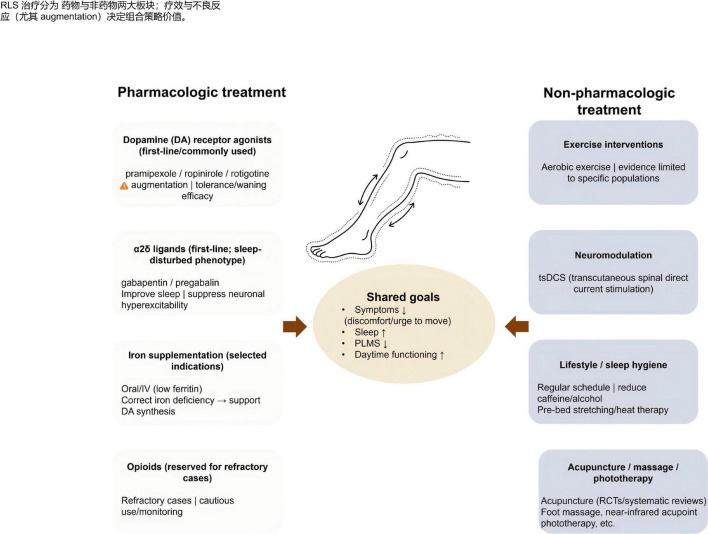
Synergistic effects of acupuncture combined with pharmacotherapy. This figure summarizes the synergistic effects of pharmacologic and non-pharmacologic treatments for restless legs syndrome (RLS). Pharmacologic treatments include DA receptor agonists (symptom relief, monitor for augmentation), α2δ ligands (improve sleep for sleep-disturbed phenotype), and iron supplementation (for iron-deficient patients). Non-pharmacologic treatments focus on exercise interventions, neuromodulation, lifestyle modifications, and acupuncture/massage/phototherapy. The shared treatment goals are: symptom relief, improved sleep, reduced periodic limb movements in sleep (PLMS), and enhanced daytime functioning. Created with Figdraw.com.

The integrative use of acupuncture together with Western pharmacotherapy can be understood through shared effects on neurotransmitter regulation, iron metabolism, and autonomic-immune interactions (Figure 4). Western drugs such as dopamine receptor agonists (e.g., pramipexole) exert their benefit by activating the DRD2/3 receptor but may lead to receptor desensitization and augmentation with prolonged use (20). α2δ calcium channel ligands such as gabapentin help regulate sleep and reduce neuronal hyperexcitability, acting on mechanisms that partially overlap with those influenced by acupuncture (39). In contrast, traditional herbal formulations, such as Shaoyao Gancao Decoction, contain components like paeoniflorin that have been shown through network pharmacology and molecular docking analyses to modulate dopaminergic, inflammatory, and iron-related molecular pathways (e.g., HMOX1, IL-17, DRD3) (40). These overlapping targets help clarify why herbal therapies may complement dopamine agonists or gabapentinoids in patients requiring multidimensional symptom control and translate into observable therapeutic synergy. A randomized controlled trial involving 46 patients demonstrated that combining acupuncture with low-dose gabapentin (300 mg/day) achieved a larger reduction in IRLSRS scores and greater improvement in Pittsburgh Sleep Quality Index (PSQI) scores compared with gabapentin alone, indicating that acupuncture may potentiate medication effects while addressing aspects of the disorder not fully responsive to pharmacotherapy (41).

Moreover, integrative treatment may reduce medication-related metabolic adverse effects. For example, chronic dopamine agonist therapy has been associated with reductions in ferritin levels, whereas iron-supportive herbal regimens such as Danggui Buxue Decoction have shown the capacity to restore iron reserves and, when used in conjunction with Western drugs, slow progression or reduce symptom instability (24). A molecular study also suggests that the combination of acupuncture and pharmacotherapy may regulate neural circuitry by influencing the expression of PTPRD, a gene implicated in locomotor activity and sleep behaviors, with acupuncture shown to upregulate PTPRD mRNA levels, and this effect amplified when paired with dopamine receptor agonists (42).

Overall, these findings illustrate that acupuncture and medication may synergistically interact across shared neurochemical, circulatory and metabolic pathways, supporting their combined use in comprehensive, mechanism-informed management of RLS.

## Clinical practice of RLS treated by acupuncture combined with medicine

In clinical settings, the integration of acupuncture with standard pharmacotherapy for RLS is starting to be increasingly used as a strategy to address both sensory-motor symptoms and accompanying sleep disruption, while minimizing the long-term risks associated with dopaminergic therapy. Unlike the mechanistic section, which focuses on how these modalities influence dopaminergic tone, iron pathways, and autonomic regulation, clinical practice emphasizes how these interventions are selected, sequenced, and adjusted based on severity, comorbidities, and individual treatment response.

Across published trials, acupuncture has been used either as a primary intervention or as an adjunct to Western pharmacotherapy. Standardized point prescriptions remain the most widely adopted approach in practice, typically incorporating lower-limb points such as ST36, SP6, BL60, and GB41 (9, 35). The clinical significance of these combinations has been confirmed in numerous RCTs, showing reductions in IRLSRS scores and improvements in sleep-related outcomes, with no significant adverse events reported (9, 35).

For patients with localized pain or marked sensory discomfort, specialized methods such as Fu’s subcutaneous needling may be considered. Case-based evidence demonstrates substantial reductions in symptom severity and longer symptom-free intervals in selected patients, suggesting a role for these techniques in refractory presentations or those intolerant to higher medication doses (43). Adjunctive modalities, such as acupoint injection at ST36 or GB41, electroacupuncture and near-infrared phototherapy, may also be strategically used. For instance, acupoint injection has been associated with immediate decreases in VAS scores in small clinical trials (8), while electroacupuncture and phototherapy have shown additional benefit in patients with end stage renan disease-associated RLS, where pharmacologic choices may be limited (15, 38).

A consistent observation across clinical studies is that combining acupuncture with medication generally produces superior outcomes compared with monotherapy, especially in patients with moderate to severe symptoms or comorbid sleep disturbance. For instance, a randomized controlled trial of 46 patients reported a substantially larger IRLSRS reduction in the acupuncture-plus-gabapentin group than in the gabapentin-only group (12.3 vs. 7.6 points), together with greater improvement in sleep quality (41). Integrative approaches may also contribute to more stable long-term control. In hemodialysis cohorts, combined acupuncture and iron supplementation maintained IRLSRS reductions several weeks beyond the treatment period (31), suggesting a potential role in preventing early recurrence in iron-deficient or secondary RLS phenotypes.

In practical terms, clinicians can consider integrating acupuncture in several ways. First, it can serve as an early adjunct in patients who continue to experience insomnia, sensory discomfort, or signs of autonomic dysregulation despite appropriate correction of iron deficiency and initiation of α2δ ligand therapy. In this context, a 2021 systematic review reported that acupuncture was associated with improvements in IRLSRS scores, although the overall methodological quality of the included trials was variable, indicating that such an approach is promising but not yet definitive (9). Second, acupuncture may have a medication-sparing role in patients who derive benefit from dopaminergic drugs but begin to show dose-limiting adverse effects or early features of augmentation. In such cases, adding acupuncture while slowly down-titrating dopaminergic therapy could help maintain symptom control while aiming to reduce long-term dopaminergic load. Third, acupuncture can act as supportive therapy in secondary RLS, particularly in complex systemic settings such as end-stage renal disease. A 2024 randomized controlled trial in hemodialysis patients demonstrated that acupuncture significantly improved IRLSRS scores and insomnia severity and favorably shifted HRV parameters, suggesting that it can provide additional benefit when pharmacologic options are constrained by comorbidities or polypharmacy (15). Lastly, from an integrated sequencing perspective, a practical, guideline-consistent approach can be conceptualized as follows: correct iron deficiency and remove exacerbating factors; initiate an α2δ ligand as first-line pharmacotherapy; introduce structured non-pharmacologic measures such as exercise, massage, or selected neuromodulation; then add acupuncture to target residual symptom burden, sleep disturbance, and autonomic imbalance; and, in carefully selected refractory cases, reserve low-dose opioids and more complex combinations, with close monitoring for safety and augmentation (3, 6, 11, 44, 45). Such an algorithm places acupuncture not as an alternative to evidence-based pharmacotherapy, but as a flexible adjunct that can enhance symptom control and potentially reduce reliance on higher-risk medication strategies.

Taken together, current evidence indicates that acupuncture can be rationally integrated with Western pharmacologic regimens to provide more stable, multidimensional symptom control. This clinical synergy aligns with both mechanistic pathways described earlier and the overall aim of your review—to outline a mechanism-informed, individualized, and practically applicable therapeutic framework for managing RLS.

## Controversies and future prospects

Despite growing interest in combining acupuncture with pharmacotherapy for RLS, several unresolved issues continue to limit widespread clinical adoption. The first controversy concerns the stability and reproducibility of therapeutic efficacy. Although numerous randomized and observational studies demonstrate benefits, some trials report notable placebo responses. For example, in a small randomized study involving 11 patients, sham acupuncture produced a 3.2-point reduction in IRLSRS, an effect size that did not significantly differ from genuine acupuncture (46), which therefore urges the need for larger, rigorously controlled trials to distinguish true physiological effects from expectancy-driven responses.

A second area of debate involves the mechanistic basis of combined therapy. Although existing evidence suggests that acupuncture influences dopaminergic signaling, iron-related pathways, and autonomic function, molecular-level data on how these effects interact with drug actions remain incomplete. For instance, how acupuncture might influence the pharmacodynamics or pharmacokinetics of dopamine agonists, such as modulation of neurotransmitter receptor sensitivity or activity of drug-metabolizing enzymes, has not been definitively characterized (9). Therefore, clarifying these molecular interactions is essential for establishing a firm biological rationale for integrative treatment.

Moreover, long-term safety remains to be further investigated as augmentation remains the primary concern associated with dopaminergic therapy. For instance, a large cohort study involving 1,799 RLS patients reported a 32.9% augmentation rate after 2 years of pramipexole use (39). Thus, adjunctive acupuncture, although it has been shown to reduce augmentation risk by lowering medication requirements or stabilizing symptom fluctuations in small studies (14), requires validation in adequately powered, long-duration trials. Safety considerations related to herbal preparations should also be interpreted cautiously, as awareness of potential hepatotoxicity or nephrotoxicity associated with certain traditional preparations remains important in integrative clinical settings.

Multidisciplinary collaboration is increasingly recommended in RLS management and is particularly relevant for combined therapy models. Research in sleep medicine has led to the development of objective assessment tools such as PSG to evaluate PLMS burden and guide treatment adjustments (47), while neurology provides expertise in dopaminergic system evaluation and pharmacologic optimization (16). Acupuncture and other non-pharmacologic modalities are generally overseen by integrative medicine or TCM departments (9), whereas rehabilitation services may support symptom reduction and sleep improvement through structured exercise interventions (36). These coordinated efforts allow for more precise identification of treatment targets and individualized therapeutic planning.

Future research could advance in three major directions. First, mechanistic studies, including molecular, neurophysiological and imaging investigations, could clarify how acupuncture modifies dopaminergic receptor expression, iron-related genes, and neural circuits, and how these changes interact with pharmacologic actions (40, 42). Second, precision-medicine approaches may allow tailoring of combined therapy based on genetic profiles; for instance, polymorphisms in MEIS1 or related loci may help predict which patients respond best to dopamine agonist-acupuncture combinations (48). Third, multicenter and long-term clinical trials are needed to validate the efficacy, safety, and durability of combined therapy. A planned international RCT involving 500 participants, designed to assess 5-year outcomes of acupuncture plus pramipexole, exemplifies the type of evidence required to guide future clinical practice (49).

Finally, standardization remains a critical challenge. Variability in acupoint selection, stimulation intensity, treatment frequency, and medication-acupuncture sequencing makes cross-study comparisons challenging, limiting their acceptance in Western practice settings. Future efforts are therefore needed for the development of unified diagnostic criteria, population-specific protocols (including pediatric and comorbid RLS), and standardized integrative treatment pathways that reflect both mechanistic insights and clinical practicality (50).

## Conclusion

Restless legs syndrome remains a clinically heterogeneous disorder in which dopaminergic dysfunction, abnormalities in iron metabolism, and heightened neural excitability interact to produce the characteristic sensory-motor symptoms and sleep disturbance. Current Western therapeutic strategies provide meaningful relief for many patients but are often limited by augmentation, incomplete symptom control, or comorbidities that restrict long-term use. In this regard, acupuncture has emerged as a plausible adjunctive option, supported by growing clinical evidence demonstrating improvements in RLS severity, sleep quality, and autonomic regulation. Although existing trials vary in size and methodology, the convergence of symptomatic benefits and mechanistic findings suggests that acupuncture may influence several of the same biological pathways targeted by Western pharmacotherapy. Therefore, integrating acupuncture with standard medical treatment offers a practical approach for patients who experience residual symptoms, medication intolerance, or sleep-related impairment. However, uncertainties remain regarding long-term safety, optimal treatment sequencing, durability of benefit, and the molecular interactions between acupuncture and pharmacologic agents.

Moving forward, progress will depend on carefully designed mechanistic studies, standardized integrative treatment protocols, and multicenter trials with adequate follow-up to clarify who benefits most from combined therapy and how it should be incorporated into routine care. By aligning Western diagnostic structure with mechanism-informed, individualized acupuncture strategies, future research may help establish a coherent and clinically workable model for integrative management of RLS.
