# Supplementary material for: Treatment of restless legs syndrome by acupuncture combined with medicine based on pathophysiological mechanism
Source: Front Med (Lausanne). 2026 May 20;13:1785644. doi: 10.3389/fmed.2026.1785644 (PMC13231477; doi:10.3389/fmed.2026.1785644)
Supplement: Supplementary file 1 [file Table_1.docx]

**Supplementary Table S1.**

**Simplified diagnostic rule set based on the CHAID decision tree model**

| **Rule** | **Clinical Decision Pathway** | **LSDS** |
| --- | --- | --- |
| ****Branch 1: Epigastric fullness = Yes**** | | |
| 1 | Epigastric fullness (Yes) → Depression or irritability (Yes) | Yes |
| 2 | Epigastric fullness (Yes) → Depression or irritability (No) → Distending pain in hypochondrium (Yes) | Yes |
| 3 | Epigastric fullness (Yes) → Depression or irritability (No) → Distending pain in hypochondrium (No) → Poor excretion of stool (No) | No |
| 4 | Epigastric fullness (Yes) → Depression or irritability (No) → Distending pain in hypochondrium (No) → Poor excretion of stool (Yes) → Excessive flatus (No) | No |
| 5 | Epigastric fullness (Yes) → Depression or irritability (No) → Distending pain in hypochondrium (No) → Poor excretion of stool (Yes) → Excessive flatus (Yes) | Yes |
| ****Branch 2: Epigastric fullness = No**** | | |
| 6 | Epigastric fullness (No) → Poor excretion of stool (Yes) → Depression or irritability (Yes) | Yes |
| 7 | Epigastric fullness (No) → Poor excretion of stool (Yes) → Depression or irritability (No) | No |
| 8 | Epigastric fullness (No) → Poor excretion of stool (No) → Diarrhea with abdominal pain (Yes) → Depression or irritability (Yes)→Excessive flatus (Yes) | Yes |
| 9 | Epigastric fullness (No) → Poor excretion of stool (No) → Diarrhea with abdominal pain (Yes) → Depression or irritability (No) | No |
| 10 | Epigastric fullness (No) → Poor excretion of stool (No) → Diarrhea with abdominal pain (Yes) → Depression or irritability (Yes)→Excessive flatus (No) | No |
| 11 | Epigastric fullness (No) → Poor excretion of stool (No) → Diarrhea with abdominal pain (No) | No |

****nstructions for clinical use:**** All symptoms are binary (present/absent) based on clinical inquiry. Follow the clinical pathway sequentially from left to right. Once a rule matches the patient's symptom pattern, LSDS classification is determined. This rule set is derived from the CHAID decision tree (Figure 5) with 11 terminal nodes.
